# Estimate of male urethritis incidences in France between 2007 and 2017 with a specific focus on *Neisseria gonorrhoeae*, *Chlamydia trachomatis*, and *Trichomonas vaginalis* infections

**DOI:** 10.1186/s12879-019-4202-1

**Published:** 2019-06-27

**Authors:** Louise Rossignol, Laurianne Feuillepain, Ndeindo Ndeikoundam Ngangro, Cécile Souty, Nelly Fournet, Yann Le Strat, Noémie Baroux, Thomas Hanslik, Florence Lot, Thierry Blanchon

**Affiliations:** 1 Réseau Sentinelles, Institut Pierre Louis d’Epidémiologie et de Santé Publique (IPLESP), UMR S 1136 Inserm Sorbonne Université, Faculté de médecine Sorbonne Université - site Saint Antoine, 27 rue Chaligny, 75571, cedex 12 Paris, France; 2 Infectious Diseases Division, Santé publique France, F-954415 Saint-Maurice, France; 3 Data Science Division, Santé publique France, F-954415 Saint-Maurice, France; 4 Université de Versailles Saint-Quentin-en-Yvelines, UVSQ, UFR de Médecine, FR-78000 Versailles, France; 5 Service de Médecine Interne, Hôpital Ambroise Paré, Assistance Publique - Hôpitaux de Paris, APHP, FR-92100, Boulogne Billancourt, France

**Keywords:** Sexually transmitted diseases, Urethritis, Male, General practice, Public health surveillance, Diagnostic techniques, Urological

## Abstract

**Background:**

In a context of increasing use of Nucleic Acid Amplification Test, diagnoses of *Neisseria gonorrhoeae* and *Chlamydia trachomatis* infections among men increased in Europe and USA since 2007. We aimed to describe trends in the incidence of male urethritis in France between 2007 and 2017.

**Methods:**

We analysed male urethritis clinical cases reported by the French GPs’ *Sentinelles* network.

**Results:**

GPs reported 1944 cases of male urethritis during the study period. The estimated annual incidence rates in men aged 15 years and older remained stable between 226 cases per 100,000 seen in 2007 and 196 in 2017 (*P* value = 0.9). A third-generation cephalosporin with macrolide or tetracycline was prescribed in 17.5% of cases in 2009 (27/154) and 32.4% in 2017 (47/145) (*P* value = 0.0327).

**Conclusions:**

The incidence rates for adult male urethritis diagnosed in primary care have remained stable since 2007 in France in contrast with the increasing trend of *Neisseria gonorrhoeae* and *Chlamydia trachomatis* infections based on microbiological surveillance. Using stable clinical definition for male urethritis seems essential to follow correctly epidemiological dynamic.

## Background

Urethritis is the most frequent syndrome of sexually transmitted infection (STI) observed in men [1, 2]. *Neisseria gonorrhoeae* (*NG*) accounts for 10 to 20% of male urethritis according to studies [3, 4]. For non-gonococcal urethritis, major urethritis pathogens are *Chlamydia trachomatis* (*CT*; 20–50% of non-gonococcal urethritis), *Mycoplasma genitalium* (15–25%), and *Trichomonas vaginalis* (1–20%). However, for 35% of male urethritis cases, pathogens cannot be documented [3–6]. Untreated or inadequately treated male urethritis can lead to serious infectious complications and infertility [1, 7]. Furthermore, STI could facilitate the transmission of human immunodeficiency virus (HIV) infection [8]. French guidelines recommend that all men who have confirmed or suspected urethritis should be tested for *NG* or *CT* [9, 10]. Guidelines for empirical treatment recommended association of ceftriaxone and doxycycline or azithromycin [9]. Since 2015, male urethritis without discharge could be treated only with doxycycline or azithromycin for empirical treatment [9, 10].

Variations in the incidence of male urethritis have been observed in France since the end of the twentieth century [11, 12]. A decrease has been described between 1989 and 1995, from 630 to 180 cases per 100,000 men aged 15 to 64 years, followed by an increase until 2003 to 325 cases per 100,000 men aged 15 to 64 years [11, 12]. These variations were correlated with sexual risk behaviours [13]. Recently, the rate of reported gonorrhoea cases among men increased in the United States since 2009 to 170.7 cases per 100,000 persons in 2016 [14]. In Europe, estimated incidences of gonorrhoea cases among men increased since 2008 to 32 per 100,000 persons in 2015 [15, 16]. The same trends among men were observed in England, with increases of 212.6 to 314.1 *CT* infections per 100,000 persons and from 46.0 to 100.5 *NG* diagnoses per 100,000 persons from 2007 to 2016 [17].

In France, mandatory reporting of bacterial STIs was stopped in 2000 and replaced by voluntarily participation of clinicians and laboratories in the surveillance program. The surveillance of male urethritis is done by a primary care surveillance system: the *Sentinelles* network.

Based on this network, the present study aims to describe the trends of acute male urethritis diagnosed by general practitioners (GPs) in France between 2007 and 2017.

## Methods

### Population and data collection

The French GP *Sentinelles* network (http://www.sentiweb.fr) is a real-time epidemiologic surveillance system inaugurated in 1984 based on about 1300 volunteer GPs (approximately 2% of all French GPs), located throughout metropolitan France [18]. Differences between *Sentinelles* GPs and all French GPs have been reported elsewhere [19]. During the study period, the mean turnover (the proportion of new reporting GPs) was 21% per year (range: 10 - 36%). The catchment area was 1 GP per 55,115 persons.

GPs reported weekly the number of male urethritis cases seen in consultation throughout the year. A case is defined by the presence of recent dysuria and/or purulent/mucoid urethral discharge in men.

Cases reported between 2007 and 2017 were included in the study. For each case, descriptive data were collected:

- From 2007 to 2017, age, presence of urethral discharge, dysuria, sexual preference, multiple sexual partners, prescription of microbiological analysis and results (for *NG*, *CT,* and *Trichomonas vaginalis*), antibiotic treatment prescription and (if prescribed) name of medication;
- From 2007 to 2015, presence of pruritus;
- From 2009 to 2017, history of STI during the past 12 months, infection with HIV, dosage and duration of the antibiotic prescribed.

### Statistical analysis

Analyses were performed to describe included cases. National incidence rates of male urethritis seen in GP consultation (per 100,000 men aged 15 years old and older) were estimated per year as the average number of cases older than 15 years notified by the *Sentinelles* GPs (adjusted for participation and geographic distribution) multiplied by the total number of GPs practicing in France and dividing incidences by yearly population size of men older than 15 years (census data) [20]. Incidences are stratified by week, and annual incidence is the sum of the incidences of the weeks in the year studied. Confidence intervals (CIs) were estimated assuming that cases reported by GPs follow a Poisson distribution.

For each year, the age-specific burden of illness was assessed with the relative illness ratio (RIR) for the following groups: 15 to 19 years old, 20 to 29 years old, 30 to 39 years old, 40 to 49 years old, 50 to 64 years old, and 65 years old and older.

Concerning microbiological analysis, numbers of available results were different according to pathogens. In France, GPs prescribe microbiological analysis, which are done in an external laboratory. GPs could specifically mention the pathogen to look for. The complex STI test was not use routinely and the result of the test for each potential causative agent was reported separately. Proportions of positive cases for *NG* or *CT* or *Trichomonas vaginalis* were calculated using cases with available information for each pathogen separately. For co-infections (*NG* and *CT*), proportions were calculated using cases with available information on both pathogens.

All analyses were performed using R software version 3.1.1.

## Results

**Fig. 1:**
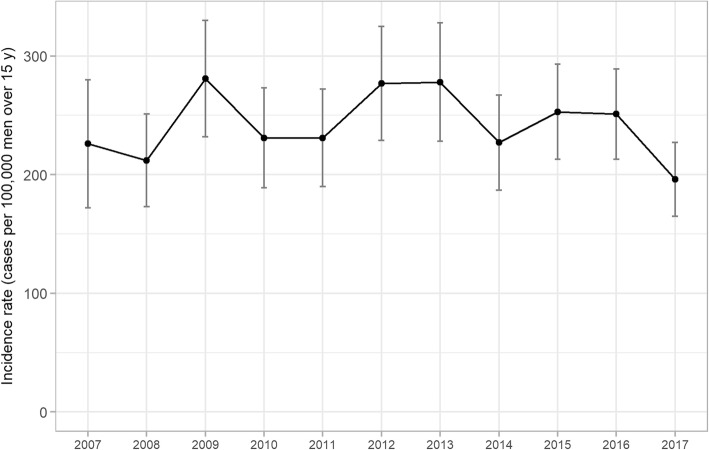
Annual incidence rates for adult male urethritis reported by general practitioners between 2007 and 2017

Between 2007 and 2017, French GPs reported 1944 male urethritis cases, among which 1733 cases were described (89.1%). The overall trend in estimated incidence rates for adult male urethritis in France remained stable during this period (Fig. 1), from 226 (CI95% [172–280]) in 2007 to 196 (CI95% [165–227]) per 100,000 men aged 15 years and older in 2017 (*P* value = 0.9). Table 1 presents male urethritis incidence rates in France by year and age group.

**Table 1:** Annual incidence rates for adult male urethritis reported by general practitioners between 2007 and 2017, by age group (95% confidence interval), *Sentinelles* network, France

Annual incidence rates [CI95]											
	2007	2008	2009	2010	2011	2012	2013	2014	2015	2016	2017
Age-group (y)											
[15,20)	177 [9–345]	36 [0–88]	177 [50–304]	207 [74–340]	158 [48–268]	309 [103–515]	220 [83–357]	121 [24–218]	159 [55–263]	272 [135–409]	157 [48–266]
[20,30)	600 [371–829]	327 [210–444]	527 [362–692]	384 [250–518]	473 [321–625]	597 [428–766]	804 [589–1019]	565 [402–728]	595 [444–746]	630 [472–788]	524 [396–652]
[30,40)	256 [118–394]	355 [234–476]	534 [358–710]	348 [224–472]	416 [283–549]	476 [319–633]	410 [248–572]	491 [338–644]	302 [190–414]	349 [236–462]	354 [244–464]
[40,50)	174 [66–282]	239 [140–338]	279 [166–392]	198 [107–289]	166 [89–243]	280 [163–397]	258 [147–369]	173 [88–258]	292 [184–400]	269 [177–361]	172 [102–242]
[50,65)	123 [48–198]	174 [101–247]	118 [58–178]	115 [50–180]	136 [73–199]	87 [27–147]	74 [25–123]	71 [28–114]	149 [85–213]	102 [51–153]	66 [29–103]
≥ 65	58 [0–125]	70 [18–122]	79 [19–139]	176 [84–268]	62 [11–113]	56 [2–110]	39 [0–78]	34 [0–69]	89 [41–137]	55 [13–97]	35 [7–63]
Total ≥ 15	**226 [172–280]**	**212 [173–251]**	**281 [232–330]**	**231 [189–273]**	**231 [190–272]**	**277 [229–325]**	**278 [228–328]**	**227 [187–267]**	**253 [213–293]**	**251 [213–289]**	**196 [165–227]**

By age group and with 95% confidence interval, *Sentinelles* network, France; Entires in boldface are total for the period 2007-2017

**Fig. 2:**
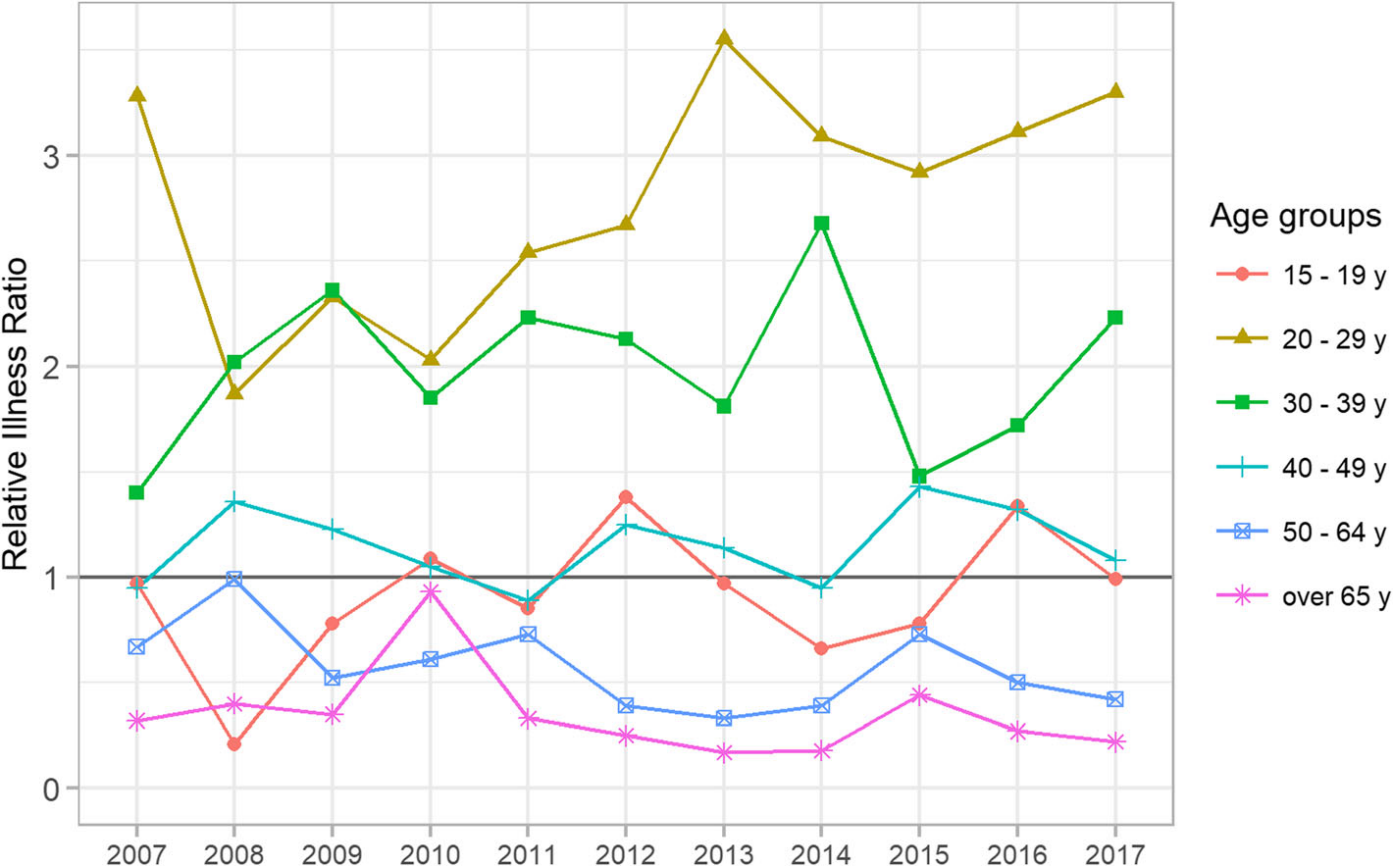
Relative illness ratio trends of male urethritis by age group from 2007 to 2017

The median age of cases was 35 years. RIRs were highest for the age groups 20–29 years and 30–39 years (Fig. 2). They were no differences between regions, in particular for Paris and its suburbs (data not shown).

The majority of the 1733 described cases had a dysuria (*n* = 1365, 79.8%) or a discharge (*n* = 1114, 65.7%), but fewer presented with pruritus (*n* = 421, 32.5%; Table 2). We observed that 37.3% of patients (*n* = 492) had multiple sexual partners and 17.2% had sex with men (*n* = 251). A previous STI history during the past 12 months was reported for 15.1% of cases (*n* = 223), and 3.5% (*n* = 43) were infected with HIV (Table 2). They were no differences in clinical signs and context from one year to the next during the study period (data not shown).

**Table 2:** Characteristics of urethritis cases reported by general practitioners between 2007 and 2017, *N* = 1733

	*n*	(%)
Age group (*missing values: 4)*		
[0,15)	8	(0.5)
[15,20)	105	(6.1)
[20,30)	612	(35.4)
[30,40)	443	(25.6)
[40,50)	279	(16.1)
[50,64)	192	(11.1)
≥ 64	90	(5.2)
Clinical signs		
Pruritus (*missing values: 57, stopped in 2015)*	421	(32.5)
Dysuria (*missing values: 22)*	1365	(79.8)
Discharge (*missing values: 38)*	1114	(65.7)
Context		
Multiple sexual partners (*missing values: 414)*	492	(37.3)
Bisexual + homosexual orientation (*missing values: 273)*	251	(17.2)
History of STI during the past 12 months (*missing values: 255)*	223	(15.1)
Known HIV infection (*missing values: 275)*	43	(3.5)

STI: sexually transmitted infection; HIV: human immunodeficiency virus; Sentinelles network, France

The trend of microbiological test prescriptions had increased between 2007 and 2017 from 65.3 to 87.2% in 2016 and 82.4% in 2017 (Table 3). Average increase for microbiological test prescriptions was 2.6% per year (CI 95% [1.6–3.6]; *P* value < 0.001). Cases were positive in 32.5% for *NG* (*n* = 234 / 720 samples analysed for *NG*), 38.0% for *CT* (*n* = 278 / 731 samples analysed for *CT*) and 2.6% for *Trichomonas vaginalis* (*n* = 16 / 623 samples analysed for *Trichomonas vaginalis*). Information on both pathogens (*NG + CT*) were available for 660 cases and 44 co-infections for *NG* and *CT* (6.7%) were diagnosed.

**Table 3:** Microbiological analysis and antibiotics prescription reported by general practitioners 2007–2017, *N* = 1733 (*n* (%))

	2007 *n* = 102		2008 *n* = 135		2009 *n* = 177		2010 *n* = 149		2011 *n* = 133		2012 *n* = 161		2013 *n* = 155		2014 *n* = 145		2015 *n* = 194		2016 *n* = 199		2017 *n* = 183		Total *N* = 1733	
Microbiological analysis prescriptions (*missing values: 52)*	66	(65.3)	73	(54.9)	103	(59.5)	104	(72.2)	90	(69.8)	104	(66.2)	116	(75.8)	107	(77.0)	144	(78.7)	163	(87.2)	150	(82.4)	**1220**	**(72.6)**
Proportion of positive *NG* cases (*missing values: 500)*	4	(14.3)	14	(26.9)	11	(24.4)	16	(30.2)	19	(38.8)	26	(45.6)	29	(42.0)	26	(33.8)	34	(34.7)	26	(27.1)	29	(30.2)	**234/720**	**(32.5)**
Proportion of positive *CT* cases (*missing values: 489)*	13	(41.9)	13	(24.1)	17	(37.0)	19	(36.5)	19	(41.3)	16	(32.0)	26	(37.7)	30	(39.5)	31	(31.3)	40	(38.1)	54	(52.4)	**278/731**	**(38.0)**
Proportion of positive *TV* cases (*missing values:597)*	2	(6.9)	2	(3.9)	0	(0)	2	(4.2)	0	(0)	6	(12)	1	(1.7)	0	(0)	3	(3.8)	0	(0)	0	(0)	**16/623**	**(2.6)**
Antibiotics prescriptions (*missing values: 267)*																								
3GC + (macrolide or tetracycline)	dnc		dnc		27	(17.5)	20	(17.2)	32	(30.5)	47	(33.8)	55	(38.4)	52	(43.0)	57	(38.3)	56	(35.7)	47	(32.4)	**393**	**(32.0)**
Macrolide	dnc		dnc		32	(20.8)	17	(14.7)	21	(20.0)	27	(19.4)	27	(18.9)	30	(24.8)	30	(20.1)	44	(28.0)	59	(40.7)	**287**	**(23.4)**
Fluoroquinolone	dnc		dnc		46	(29.9)	32	(27.6)	17	(16.2)	22	(15.8)	14	(9.8)	15	(12.4)	17	(11.4)	17	(10.8)	13	(9.0)	**193**	**(15.7)**
Tetracycline	dnc		dnc		20	(13.0)	16	(13.8)	8	(7.6)	13	(9.4)	16	(11.2)	3	(2.5)	11	(7.4)	14	(8.9)	10	(6.9)	**111**	**(9.0)**
Others	dnc		dnc		29	(18.8)	31	(26.7)	27	(25.7)	30	(21.6)	31	(21.7)	21	(17.3)	34	(22.8)	26	(16.3)	16	(11.0)	**245**	**(19.9)**

dnc, Data not collected; 3GC: third-generation cephalosporin; others: aminoside/aminoside +3GC/aminoside + macrolide/aminoside + tetracycline/3GC/3GC + fluoroquinolone/3GC + fluoroquinolone + cycline/3GC + macrolide + fluoroquinolone/3GC + macrolide + imidazole/3GC + macrolide + tetracycline/3GCG + penicillin/fosfomycin/imidazole/macrolide +2GC/macrolide + fluoroquinolone/macrolide + imidazole/macrolide + tetracycline/nitrofurane/penicillin/penicillin-clavulanic acid/penicillin-clavulanic acid + fluoroquinolone/penicillin-clavulanic acid + macrolide/quinolone/streptogramines/trimethoprim-sulfamethoxazole/trimethoprim-sulfamethoxazole + macrolide/tetracycline + fluoroquinolone/tetracycline + imidazole; *NG*, *Neisseria gonorrhoeae; CT*, *Chlamydia trachomatis; Sentinelles network, France; Entires in boldface are total for the period 2007-2017*

Antibiotics were reported in the questionnaire for 82.2% of cases between 2009 and 2017 (*n* = 1229/1496). Third-generation cephalosporin (with macrolide or tetracycline) was prescribed for 32.0% of cases (*n* = 393) between 2009 and 2017. This proportion progressively increased from 17.5% (*n* = 27) in 2009 to 43.0% (*n* = 52) in 2014 and then decreased to 32.4% (*n* = 47) in 2017 (Table 3). Average increase for third-generation cephalosporin (with macrolide or tetracycline) prescription was 2.3% per year (CI 95% [0.3–4.4]; *P* value = 0.0327). A prescription of fluoroquinolone alone decreased from 29.9% (*n* = 46) in 2009 to 9.0% (*n* = 13) in 2017 (Table 3). Average decrease for fluoroquinolone alone prescription was 2.5% per year (CI 95% [1.2–3.7]; P value = 0.002).

## Discussion

The overall trend in the incidence rates for adult male urethritis seen by GPs has remained stable since 2007 in France. The most affected age groups were 20–29 and 30–39 years. GPs’ adherence with STI treatment recommendations improved during the study period.

The stability of the incidence rate of male urethritis in France does not seem to be in alignment with European and American STI trends [14–16, 21, 22]. Laboratory-confirmed gonorrhoea cases in women and men increased in Europe, the United Kingdom, and the United States in the past decade [14–17]. The increase in gonorrhoea cases largely concerned the population of men who have sex with men (MSM) [15, 16]. For *CT* infection, the U.S. rate of reported cases increased between 2000 and 2016 [22], and European data showed an increase between 2004 and 2009 and a stability since this date [15, 21]. Most *CT* infections concerned young adult women and heterosexuals [15, 21]. In France, laboratory network and RésIST network (STI clinics, dermatological hospital consultation units, infectious illness consultation units, internal medicine or private medical practices) reported an increase for the number of rectal lymphogranuloma venereum and gonorrhoea of 26 and 127% respectively, between 2014 and 2016 [23]. In the same period, they also reported an increase for the number of early syphilis in MSM of 35% [23]. After two decades of increase, the number of chlamydia infections decreased between 2015 and 2016 (− 7%) [23]. In South Africa, recent data have shown a stable gonorrhoea and chlamydia prevalence over 1990–2017 in both sexes [24]. However, the American and European surveillance systems for STI vary a lot between countries, with some systematically using a definition of STI cases with laboratory criteria and including symptomatic and asymptomatic cases. Reporting sources usually could include STI clinics, private physician, emergency and other hospital room, or family planning. It is difficult to compare etiological results that include asymptomatic cases diagnosed by GPs and specialists with the trends in the incidence rates for adult male urethritis in French primary care when restricted to a syndromic and symptomatic definition. Moreover, national surveillance data based on STI clinics include high-risk groups that are non-comparable to the general population visiting GPs [14, 17, 22].

Elsewhere during the 2000s, introduction of Nucleic Acid Amplification Test (NAAT) and its diffusion worldwide probably had a direct impact on the number of positive diagnoses, in particular for asymptomatic STI. As noticed in the 2013 European surveillance report, this new diagnostic method and the evolution in testing policies have directly affected STI surveillance throughout the world, and it is hard to determine the cause of the last increase in *NG* and *CT* infection cases (i.e., real increase, improvement in screening coverage, or improvement in diagnostic tools) [15]. With regard to *NG* infection, recent international guidelines recommended testing urethral, pharyngeal, and rectal sites among MSM using NAAT for routine laboratory screening [25–27]. Among MSM attending the Melbourne Sexual Health Centre, a third of men with urethral gonorrhoea had concurrent pharyngeal gonorrhoea and two third had rectal gonorrhoea [28]. However, pharyngeal and rectal sites may increase the diagnoses of asymptomatic *NG* infection. This could have also increased the number of *NG* cases in Europe and the United States. In addition, *CT* case detection had been improving with better detection tools [15]. Using NAAT for *CT* and *NG* detection had been promoted by different national and international guidelines, in particular for routine screening. For male urethritis, guideline have not changed during the study period: all men who have confirmed or suspected urethritis should be tested for *NG* or *CT* [9, 10, 27]. If patient had a purulent/mucoid urethral discharge, culture on urethral swab specimens is recommended looking for *NG* and NAATs on urine specimens for *CT* [10]. If not, NAATs on urine specimens are recommended looking for *NG* and *CT* [10]*.* Nowadays, most of countries recommend testing all young women and men or specific populations, such as those with as multiple sexual partners, MSM, pregnant women, and vulnerable populations [26, 27, 29]. In France, *CT* screening has been recommended since 2003 for young women (< 25 years) and men (< 30 years) who visit free STI clinics or family-planning centres. In 2018, this recommendation was expanded to all health services for groups at risk [30].

With regard to male urethritis, microbiological tests are useful for evaluating *NG* antimicrobial resistance, which could lead to untreatable gonorrhoea infections [31]. Its surveillance is necessary to guide empirical treatment [32, 33]. Concerning French GP practice, the proportion of third-generation cephalosporin associated with a macrolide or a tetracycline increased by 146% and reached 43% in 2014, but it decreased to 32.4% in 2017, although national guidelines recommend third-generation cephalosporin use with azithromycin or doxycycline [9]. Fluoroquinolone alone still represents an important proportion of the prescriptions by GPs (9.0% in 2017), despite the high proportion of resistant gonococcal strains in France [33]. Improvement in GPs’ practice has also been reported in England, where prescriptions of ciprofloxacin have decreased from 42 to 20% of prescriptions between 2007 and 2011, and cephalosporin prescriptions have increased and reached 35% in 2011 [34]. A recent study in Ontario assessed adherence with first-line gonorrhoea treatment recommendations in the context of several updated guidelines [35]. During the study period (2006–2014), two new recommendations were introduced (in 2008 and 2011) due to the evolution of antimicrobial resistance. This study highlighted the difficulties in the dissemination and the implementation of new guidelines.

The major strength of our study is its use of the same clinical case definition for male urethritis over the study period, which allows for comparisons over time. Moreover, syndromic surveillance may not be affected by changes in testing policies and improvement in diagnostic tools. However, our clinical definition did not include data on inflammatory reaction in urethra (no biological confirmation needed). And this surveillance is blinded concerning asymptomatic *NG* or *CT* infections or other infections such as HIV or syphilis. For male urethritis cases included in the study, data collected on *Mycoplasma genitalium* do not allow to present analysis. This is a limitation according to the significant proportion of male urethritis cases caused by this pathogen. Other limitations are the absence of syndromic surveillance for women. Basing our system on general practice and not on STI clinics does not permit estimation of the incidence in high-risk transmission groups such as MSM. Concerning sexual behaviour, there was no question specifying the exposure period for both items “having multiple sex partners” and “sex with other men”. This represent a limitation in the interpretation of the data.

## Conclusions

Even if most of the other settings (such as STI clinics) reported a increase in the *NG* and *CT* cases across Europe and United States, data from French GPs show a stable trend in the incidence of male urethritis rate. Because of the variety of reporting source, these two settings cannot be compared. Showing a stable trend in the incidence of male urethritis rate in France, this work highlights the need to use clinical stable definitions to follow correctly epidemiological dynamics of such a disease, especially in a context of improvement of laboratory diagnostic techniques. Changes in testing policies could impact STI trends, especially on microbiological surveillance, and should be evaluated to better understand the epidemiological evolution of STI.

## Data Availability

The datasets supporting the conclusions of this article are available for the *Sentinelles* network on our websites (http://www.sentiweb.fr) and specific data could be request with the form available here: https://www.sentiweb.fr/?page=request.
